# Death from Functional Nervous Disease

**Published:** 1899-04-15

**Authors:** 


### DEATH FROM FUNCTIONAL NERVOUS DISEASE.

The very interesting paper read by Dr. Maguire before the last meeting of the Medical Society on Death from Functional Nervous Disease, however little convincing it may be in regard to the particular thesis which he endeavoured to maintain, at least serves to emphasise a fact which is far too often overlooked, namely, that the post-mortem room in many cases tells us wondrous little as to the final cause of death in the cases that appear on the table. That one case has a disease of the heart and another a tumour of the brain is plain sailing, and that in both these maladies death is an event not greatly to be surprised at is fairly obvious; but why the patient died yesterday rather than the day before, and, indeed, why he died at all, seeing how long the condition met with must have continued in very much the same degree of severity, is a matter in regard to which it is to be feared that our investigations with the scalpel often throw no light. Dr. Maguire would have us believe that cases occur in which death is due to mere exhaustion of the grey nerve matter. In such cases the only system at fault is the highest nervous system, and he considers that the death is due to exhaustion of the grey nervous matter which controls all the other functions. Among the cases that he mentioned was one of a young woman who was brought to St. Mary's Hospital in an unconscious state, in which she lay for four days before death, presenting no sign of disease except the unconsciousness. There was no trace of poisoning, and nothing was found post-mortem. She had been greatly troubled before the attack. Another case was that of a medical student who had lived very sparingly and had had difficulty in completing his curriculum. Shortly after passing his examinations he became unconscious, but presented no other physical signs of disease. In three months he gradually recovered. He then began practice, and married, but in three years' time was taken ill again with the same symptoms and died. Nothing was found at the post-mortem examination.

That such cases occur will, we think, be generally admitted. What they mean is another matter. The word "functional" is one which we must hesitate to accept as meaning "inorganic," or indeed as meaning more than that no gross lesion is discoverable. If we are to admit that people, otherwise healthy, can die merely because their grey nerve matter is "tired," we must admit also that people who are already actually diseased must be still more liable to the same accident—and perhaps they are. There are many ups and downs in the course of chronic exhausting diseases, many "good days" and "bad days" which certainly are hard to explain or to connect with any variation in the progress of the disease itself, and such variations would be easily and apparently reasonably enough explained if a superadded "exhaustion of grey nerve matter" were admissible. We doubt it. It seems probable that all these varying conditions and degrees of vitality are reducible to terms of nutrition and of toxæmia, but at least it is worth pointing out that if we are to accept "exhaustion of grey matter" as an efficient cause of death in the apparently healthy, still more must we admit it as a sufficient explanation of the not uncommon instances in which patients quietly slip out from life apparently from a deficient power of living rather than from any aggravation of the disease from which they are known to have been suffering.

